# Volatile Aroma Compounds in Various Brewed Green Teas

**DOI:** 10.3390/molecules180810024

**Published:** 2013-08-20

**Authors:** Jeehyun Lee, Delores H. Chambers, Edgar Chambers IV, Koushik Adhikari, Youngmo Yoon

**Affiliations:** 1 Department of Food Science and Nutrition, Pusan National University, 30 Jangjeon-Dong, Geumjeoung-Ku, Busan 609 735, Korea; E-Mail: jeehyunlee@pusan.ac.kr; 2 Sensory Analysis Center, Kansas State University, Department of Human Nutrition, Manhattan, KS 66506-1407, USA; E-Mails: eciv@ksu.edu (E.C.); koushik@ksu.edu (K.A.); 3 Sensient Flavors LLC, 5600 West Raymond St., Indianapolis, IN 46241, USA; E-Mail: youngmo.yoon@sensient.com

**Keywords:** green tea, aroma, volatiles, GC-MS, HS-SPME

## Abstract

This study identifies and semi-quantifies aroma volatiles in brewed green tea samples. The objectives of this study were to identify using a gas chromatograph-mass spectrometer (GC-MS) paired with a headspace solid-phase micro-extraction (HS-SPME) the common volatile compounds that may be responsible for aroma/flavor of the brewed liquor of a range of green tea samples from various countries as consumed and to determine if green teas from the same region have similarities in volatile composition when green tea samples are prepared for consumption. Twenty-four green tea samples from eight different countries were brewed as recommended for consumer brewing. The aroma volatiles were extracted by HS-SPME, separated on a gas chromatograph and identified using a mass spectrometer. Thirty-eight compounds were identified and the concentrations were semi-quantified. The concentrations were lower than those reported by other researchers, probably because this research examined headspace volatiles from brewed tea rather than solvent extraction of leaves. No relationship to country of origin was found, which indicates that other factors have a greater influence than country of origin on aroma.

## 1. Introduction

Aroma, flavor and appearance are important aspects of the evaluation of green tea [1] and other products such as spice tea [2]. Many researchers have studied green tea for its aromatic volatile compounds to understand the aroma characteristics of green teas [3,4,5,6,7,8,9,10,11,12]. However, in most prior research a limited number of green tea samples were used and the sample preparation did not reflect how green tea commonly is prepared by consumers [13,14,15]. In one study [12], for example, 60 g of green tea were brewed in 1 L of 60 °C water for 5 min; the green tea liquor then was steam distilled and concentrated to 10 µL for gas-chromatographic analysis. Typical brewing methods recommend using 2 g of green tea in 55 mL at 60–70 °C for 1–2 min [16]. 

Various green teas are available to consumers that represent different processing methods, harvest times, plant varieties and growing regions, all of which may contribute to different aroma characteristics in each tea [13]. For example, Ryu [17] used a solid-phase micro-extraction (SPME) method and found that volatile compounds were lower in teas grown in lower temperatures than ones picked from the same fields a year later when temperatures were warmer. Studying aromatic volatile compounds from brewed green tea liquor can help researchers to understand the composition of tea brewed as consumers actually would consume it. Recently it was suggested that sensory quality of Japanese green tea could be classified as either high or low using gas chromatography-mass spectroscopy (GC-MS) techniques on actual brewed samples [18].

Volatile compounds in green teas extracted with solvents have been studied extensively using gas chromatography-mass spectrometry (GC-MS) to understand the differences in green tea aromatic components [19]. More than 600 volatile compounds have been identified through extensive research on green tea [20]. Researchers have studied green tea aroma in *Hojicha* (roasted green tea from Japan) [21], *Bach-Mao* tea (Vietnamese green tea) [22], *Sencha*-type spring green teas (steamed green tea from Japan) [23], *Kabusecha* (a Japanese green tea made from tea leaves grown in the shade) and *Sencha* (steam-processed green tea from Japan) [5]. Choi [7] identified 41 volatile compounds from four commercial Korean green teas; two samples were steam-processed and two were roast-processed. Kumazawa and Masuda [11] used GC-MS and found 54 compounds from *Kamairi-cha* (a Japanese tea; roast-processed), *Longjing* tea (a Chinese tea; roast-processed) and *Sencha.* Eighteen volatile compounds were found in 23 Chinese green teas and 1-penten-3-ol, linalool, terpineol, citral, citronellol, nerol, geraniol and others commonly were present [24]. In another study 97 compounds common to teas from two regions in China were found and the authors were able to discriminate among the teas of two regions using those compounds measured by GC-MS analysis [25]. A review of recent studies on volatile compounds in tea did not indicate any studies had been conducted using brewed green tea and headspace analysis [26].

SPME is a type of a headspace sampling, which studies the volatile compounds in the space above a sample in a sealed container [27]. Thus, using SPME with green tea brewed as a consumer typically would brew it may help understand the volatiles that would be in the aroma of green as consumers drink it. SPME is a convenient technique because there is no complicated sample preparation and it is environmentally friendly because it does not require any solvents. The objectives of this study were to use GC-MS paired with SPME to (a) identify common volatile compounds in the brewed liquor of a wide range of green tea samples from various countries and (b) determine if green teas from different regions can be discriminated based on volatile composition in samples prepared for normal consumption.

## 2. Results and Discussion

### 2.1. Volatile Compounds in Prepared Green Teas

Thirty-eight aroma volatile compounds were tentatively identified from the 24 green tea samples (Table 1). Table 1 also provides information on potential flavors associated with those compounds from prior literature. Because sensory characteristics were not measured in this study, those characteristics merely are examples of potential associations and what might be expected given the chemical composition. Thus, such information must be considered only a tentative association [28].

**Table 1:** 39 volatile compounds identified in 24 green tea samples and their odor characteristics reported as the literature.

Volatile Compounds	Odor characteristics	Reference
*Aliphatic Alcohols*		
1-Penten-3-ol	Butter, mild, green	[29]
1-Pentanol	Somewhat sweet, balsamic	[29]
2-Penten-1-ol	Jasmine, green, plastic, rubber	[29,30]
(*Z*)-3-Hexen-1-ol		
Linalool	Bergamot oil, French lavender	[29]
Geraniol	Pleasant geranium	[29]
*Aromatic Alcohols*		
Benzeneethanol	Floral, rose	[29]
1-α-Terpineol	Lilac	[29]
2-Methoxy-4-methylphenol		
2,6-Dimethyl-cyclohexanol		
*Aliphatic Aldehydes*		
3-Methyl-butanal	Unpleasant	[29]
Pentanal	Pungent, almond, malt	[29,30]
Hexanal	Fruity	[29]
*cis*-3-Hexenal	herbal apple, green leafy	[29]
Nonanal	Fatty, citrus, green	[29,30]
1H-Pyrrole-2-carboxaldehyde		
*Aromatic Aldehydes*		
Benzaldehyde	Bitter almond	[29]
Benzeneacetaldehyde	Hyacinth, lilac	[29]
*Other Aromatic Compounds*		
Toluene	Paint	[30]
1,4-Dimethoxybenzene	Aromatic ether, sweet clover	[29]
1,4-Bis(1,1-dimethylethyl)-benzene		
Styrene	Penetrating , balsamic, gasoline	[29,30]
2-Hydroxy methyl ester benzoic acid		
*Ketones*		
3,5-Octadien-2-one		
Jasmone	Jasmine	[29]
α-Ionone	Woody, violet	[29]
β-Ionone	Woody	[29]
2-Methyl-5-(1-methylethenyl)-2-cyclohexen-1-one (=Carvone)	Mint	[30,29]
*Furans*		
Tetrahydro-2,2,5,5-tetramethyl furan		
4-Methyl-2-propyl furan		
N-Furfuryl adenine		
*Pyridine*		
3-Butyl-1-oxide-pyridine		
*Pyrazine*		
3-Ethyl-2,5-dimethyl-pyrazine		
*Furanone*		
5,6,7,7a-Tetra 2(4)-benzofuranone		
*Acids*		
Benzoic acid	Aromatic acid	
2-Hydroxy methyl ester benzoic acid		
Nonanoic acid	Fatty, green	[29,30]
*Ester*		
Isopropyl myristate	Odorless	[29]

Of the 39 volatile compounds identified and quantified in this study, 15 volatiles present in more than four samples are shown in Table 2 and the remaining compounds, found in only a few samples, are shown in Table 3. Some of the compounds (e.g., geraniol, linalool, indole, and jasmine) are similar to those found in brewed Japanese green tea samples [18]. Because the focus of the study was to determine the presence of compounds and the distribution of those compounds in green teas grown around the world, the semi-quantification method using an internal standard was employed. Thus, differences in concentrations among tea samples were not determined statistically for this research. Standard deviations were typical for studies of this type, usually between 10 and 50% of the mean value, suggesting low to moderate variation.

#### 2.1.1. Volatile Concentration

The concentrations of the semi-quantified compounds in this study were much lower than reported elsewhere. This probably is because of the differences in sample brewing and extraction. Extracting volatile compounds using organic solvents, as is typical in previous literature, generally leads to the extraction of most of the volatile compounds. Headspace analysis of green tea that has been brewed using a typical consumer preparation method is not efficient in terms of solubilization of many of the non-polar volatiles. However, although it is less efficient, headspace analysis may be more useful in terms of understanding the compounds that are present when consumers smell/drink tea and decide if they like or don’t like it.

**Table 2:** Average concentration of 14 common volatile compounds identified in the brewed green tea samples (ng/kg) ^a,b^.

Sample	1-Penten -3-ol	2-Penten -1-ol	Linalool	Geraniol	Benzeneethanol	2,6-Di-methyl-cyclo- hexanol	Pentanal	Hexanal	Nonanal	Benz aldehyde	Toluene	2-Hydroxy-3-methyl benzoic acid	Styrene	Jasmone	β-Ionone
Kovats Retention Index	760	859	1232	1420	1261	1259	780	889	1238	1281		1359	1006	1589	1691
*Africa*															
Kenya 1	0.56	1.07	1.35	-	-	-	0.76	-	1.32	0.45	-	-	-	-	-
Kenya 2	0.22	0.52	3.21	0.34	-	-	-	0.61	-	0.51	-	-	-	-	0.31
Tanzania	- ^c^	-	1.56	-	-	-	-	0.44	-	0.54	-	-	-	-	-
*Southeast Asia and Indian Subcontinent*															
India	-	-	0.96	-	-	-	-	-	0.77	-	-	-	-	-	-
Sri Lanka 1	-	0.65	2.35	-	-	-	0.39	0.96	0.32	-	-	0.72	-	-	0.61
Sri Lanka 2	-	-	0.83	-	-	-	-	-	1.17	-	-	-	-	-	-
Vietnam	0.80	0.64	0.74	-	-	0.33	-	0.61	-	0.74	-	-	-	-	0.80
*Northeast Asia*														
China 1	-	-	-	-	-	-	-	-	0.38	-	-	0.43	-	-	-
China 2	0.14	-	-	-	-	0.48	-	0.37	0.22	-	-	-	-	-	0.62
China 3	-	-	0.45	0.94	-	-	-	0.30	0.38	-	0.42	-	-	0.31	0.22
China 4	-	-	1.34	-	-	-	-	0.28	-	-	-	0.52	-	-	-
China 5	-	-	0.58	0.43	0.88	-	-	0.53	0.57	0.36	3.04	-	-	0.66	-
China 6	0.29	1.52	0.45	-	-	-	0.92	0.73	0.61	0.65	-	-	-	-	-
China 7	-	-	0.55	0.42	0.42	-	-	0.53	0.49	0.41	-	-	-	0.50	-
Japan 1	-	-	0.50	-	-	0.66	-	0.54	0.48	-	0.86	-	0.25		1.21
Japan 2	-	-	0.27	-	-	-	-	-	0.41	-	0.35	-	0.21	-	-
Japan 3	-	-	0.27	0.23	1.18	-	0.22	0.29	0.36	0.40	3.40	-	-	1.13	0.25
Japan 4	-	-	-	-	-	-	-	-	-	-	-	-	-	-	-
Japan 5	-	-	0.37	-	-	0.50	0.52	0.45	-	-	-	-	0.22	-	0.97
Japan 6	-	0.48	-	-	-	-	-	0.36	0.34	-	-	0.55	-	-	0.53
Korea 1	-	-	0.32	-	0.54	-	-	0.45	-	-	-	-	-	-	-
Korea 2	-	-	0.39	-	-	0.40	0.31	0.43	0.37	0.27	-	-	0.18	0.52	-
Korea 3	0.32	1.07	2.34	0.84	0.54	-	-	0.70	0.47	0.84	-	-	-	0.55	0.76
Korea 4	-		0.98	0.60	0.58	-	-	0.96	0.62	0.33	0.70	-	-	1.06	-

^a^ The volatiles were separated on an Rtx^®^-5 (Crossbond^®^ 5% diphenyl-95% dimethyl polysiloxane; 30 m length × 0.25 mm internal diameter × 0.25 μm film thickness) capillary column (Restek; Bellefonte, PA, USA); ^b^ Concentration is reported in nanogram per kilogram of green tea when 1 kg of green tea is brewed in 25 kL of 70 °C water for 2 min; ^c^ - = not detected.

**Table 3:** Average concentration of 24 uncommon volatile compounds identified in the brewed green tea samples (ng/kg) ^a,b^.

Sample	1-Pentanol	(Z)-3-Hexen-1-ol	1-α-Terpineol	2-Methoxy-4-methylphenol	3-Methyl-butanal	2,6-Dimethyl-cyclo hexanol	*cis* -3-Hexenal	1H-Pyrrole-2-carboxaldehyde	Benzene-acetaldehyde	1,4-Dimethoxy benzene	1,4-Bis(1,1-dimethylethyl)-benzene	3,5-Octadien-2-one
*Africa*												
Kenya 1												
Kenya 2						0.21	2.57		1.21			
Tanzania			0.48									
*Southeast Asia and Indian Subcontinent*												
India												
Sri Lanka 1			0.45									
Sri Lanka 2												
Vietnam				1.0						0.35		
*Northeast Asia*												
China 1												
China 2												
China 3												
China 4			0.27									
China 5											0.40	
China 6												
China 7		0.33										
Japan 1												
Japan 2												
Japan 3												
Japan 4												
Japan 5												
Japan 6												
Korea 1												
Korea 2								0.56				0.58
Korea 3						0.33	5.34		2.69		0.29	
Korea 4	0.27	0.47										
*Africa*												
Kenya 1												
Kenya 2										0.93		
Tanzania		0.67										
*Southeast Asia and Indian Subcontinent*												
India												
Sri Lanka 1				0.60					11.58			
Sri Lanka 2												
Vietnam												
*Northeast Asia*												
China 1			0.53									
China 2	0.23							0.22				
China 3						0.29						
China 4												0.23
China 5					0.30							
China 6												
China 7												
Japan 1	0.38							0.43				
Japan 2												
Japan 3					0.18							
Japan 4			0.35									
Japan 5												
Japan 6			1.21									
Korea 1												
Korea 2												
Korea 3							0.46			1.94		
Korea 4						0.26					0.85	

^a^ The volatiles were separated on an Rtx^®^-5 (Crossbond^®^ 5% diphenyl-95% dimethyl polysiloxane; 30m length × 0.25 mm internal diameter × 0.25 μm film thickness) capillary column (Restek; Bellefonte, PA, USA); ^b^ Concentration is reported in nanogram per kilogram of green tea when 1 kg of green tea is brewed in 25 kL of 70 °C water for 2 min

In this research, the concentrations of most volatile compounds were lower than sensory thresholds reported in other work. The threshold values in the literature most likely varied because different measuring methods were employed and sensitivity of assessors vary [31]. Also the thresholds in the literature were measured at room temperature of 21 ± 1 °C [32] or not specified [31] whereas green tea is consumed at a higher temperature. This higher drinking temperature may result in lowering thresholds of aroma compounds. Another aspect to consider is that thresholds in the literature were evaluated in a single compound dilution while volatile compounds in green tea do not work in solitude. The interrelationships of the compounds may result in an aroma or flavor even when the individual compounds are at too low a concentration [28].

#### 2.1.2. Compounds Present

Twenty green tea samples contained linalool, which has a “light, refreshing, floral-woody with a faintly citrusy note” [33]. Concentrations of linalool in final products range from 2 to 10 μg/L in candy and beverages and could be as high as 40 μg/L in meat products [33]. Concentrations of linalool in the green tea samples in this study were much lower and ranged from 0.27 ng/kg to 3.34 ng/kg. These were 1,000 times lower than orthonasal and retronasal thresholds, which are 5–6 μg/L and 1.5 μg/L in water, respectively [31].

Seventeen green tea samples had nonanal and its concentration varied from 0.22 ng/kg to 1.32 ng/kg. Nonanal has a tallowy and fruity odor [31] and its typical concentrations in final products range from 0.2 to 6 μg/L [33]. Nonanal is widely reported in green tea [12,34,35]. However, a direct comparison of concentrations between the samples in previous research and our green tea samples is not possible because only relative quantities have been reported in the form of peak area percentages. The threshold of nonanal is ~1 μg/L for orthonasal evaluation [31].

Eleven green tea samples had benzaldehyde at concentrations lower than 1 ng/kg. Benzaldehyde has a dominantly sweet aroma of freshly crushed almonds; it typically is used in artificial cherry flavorings. The typical concentrations in final products usually range from 150 to 160 μg/L [33]. Benzaldehyde has been identified in green tea by many researchers [7,8,12,21,34,36] evaluating samples from the Azores, China, Japan, Korea and Taiwan. 

Ten samples had β-ionone in concentrations ranging from 0.25 ng/kg to 1.21 ng/kg. β-Ionone has a violet-like odor and can be detected at concentrations as low as 0.007 μg/L [31] in water. Typical concentrations in final products range from 1 to 10 μg/L [33]. β-Ionone is commonly found in green teas [7,8,12,24,37,38] with peak areas ranging from 1.36 to 4.49% in Japanese green teas [12].

Hexanal was found in eight samples at concentrations lower than 1 ng/kg. Other researchers found hexanal in green teas in trace or small amounts [7,12,36]. The aroma of hexanal has been described as being similar to freshly cut grass and unripe fruits in extreme dilution. However, hexanal has been shown to change character completely, from green to beany, which combined with other compounds [39]. Its concentrations in final food products generally is given as between 1 μg/L and 5 μg/L [33]. The threshold of hexanal ranged from 4.5 μg/L to 50 μg/L for orthonasal evaluation and from 10.5 μg/L to 16 μg/L for retronasal evaluation [31], which is at least 10^4^ times higher than the concentrations found in any of the samples in our study. Even though the concentrations are lower than threshold, hexanal may contribute to aroma in combination with other volatile compounds [39] as might occur in green tea.

Geraniol, a terpene alcohol, was present in seven samples at concentrations below 1 ng/kg. It has been found in green tea by other researchers [7,8,12,36,37,38] and is a colorless, oily liquid with a flowery, rose-like aroma [33]. The orthonasal threshold reported for geraniol varies from 5 to 75 μg/L in water [31] and concentrations of geraniol in the samples in this study were well below the reported threshold values.

Seven green teas had jasmone in concentrations that ranged from 0.31 ng/kg to 1.13 ng/kg. Jasmone has a floral odor [33] and has been found in many green tea samples [7,12,34,38]. Korean green teas manufactured from leaves picked in April were found to have more jasmone than the samples harvested in June [7]. A similar trend was found in our study in that Korea 2, 3 and 4 (all of which were picked in April) had jasmone but the compound was not detected in the Korea 1 sample (which was picked in May). Jasmone was not detected in most of the green tea samples in our study including Japan 5 (*Gyokuro*). Jasmone has been reported in *Gyokuro* having 4.16% of volatile compounds extracted [40].

Seven samples had 2-penten-1-ol at various concentrations ranging from 0.48 ng/kg to 1.52 ng/kg. Research [7,8] has found *cis*-2-penten-1-ol in green tea samples and have classified it as an off-flavor. However, direct comparison of the concentrations was not possible as only peak area was reported in those studies [7,8]. The compound has been reported at a much higher concentration of 1.35 mg/kg in green tea than in our findings [41]. This difference probably is because of the variation in sample extraction. In that study [41] the sample was prepared by solvent extraction directly from green tea whereas in our study the tea samples were brewed in water.

1-Penten-3-ol was in six samples at a level of less than 1 ng/kg. It has been described as having a powerful, grass-green and very diffusive odor but the recognition threshold for this compound was not reported [33]. It has been found that green tea samples and green teas made with leaves harvested in June, when compared to those harvested in April, had higher levels of 1-penten-3-ol [7]. In a later study it was found that steam-processed samples had twice the amount of 1-penten-3-ol compared toroast-processed samples [8]. Contrary to those reports, 1-penten-3-ol was to be prevalent in stored and/or lower quality green teas in one study [42]. In this study, steam-processed green tea samples from Japan did not contain 1-penten-3-ol, although other C5 compounds (pentanal or 2-penten-1-ol) were present. Our research did show that 1-penten-3-ol was present in various Chinese and Korean samples including Korea 3, which was made from leaves collected during the first picking in April and roast-processed. At higher levels, 1-Penten-3-ol can be considered an off-flavor because it has a pungent odor, but the amounts found in our samples were well below those levels [31].

Benzeneethanol was present in six green tea samples. Concentrations in these samples were much lower than threshold, which is 1,000 μg/L for orthonasal evaluation and 45 μg/L for retronasal evaluation [31]. The compound has been reported to have a floral rose odor [29].

Six samples had toluene, which has a sweet-grassy odor [33]. Toluene was reported by a limited number of researchers studying green tea [21,37]. Samples in this study had a wide range of concentrations from 0.35 to 3.40 ng/kg.

Six samples in the study had pentanal, which typically has a woody, vanilla-like, nutty odor [37]. Pentanal has been detected in green tea when the sample was extracted using the dynamic headspace technique but it was not detected when it was extracted by simultaneous distillation-extraction technique [35]. Because pentanal is one of the first compounds to emerge from a GC column during separation, it could be an actual aromatic in the tea or may be introduced with other compounds from the column and/or be trapped residue.

Five green tea samples had 2,6-dimethyl-cyclohexanol. It was detected in green tea when the sample was extracted using a dynamic headspace technique but was not found when extracted by the simultaneous distillation-extraction technique [35]. In this study, the concentration ranged from 0.33–0.66 ng/kg, but comparison of these results with other literature reports is not possible because previous researchers only reported percentage peak areas. Threshold information also was not found for 2,6-dimethyl-cyclohexanol. Styrene and 2-Hydroxy-3-methyl benzoic acid were detected in four samples each.

#### 2.1.3. Composition Based on Geographic Area

Green teas produced in the Africa region commonly had 1-penten-3-ol, 2-penten-1-ol, linalool, hexanal, and benzaldehyde. These green teas may provide green, floral, fruity and nutty aroma during infusion and consumption. No other information on volatile compounds of green tea from Africa was found. 

Green teas from Southeast Asia and the Indian subcontinent generally had linalool and nonanal and half of the samples had 2-penten-1-ol, hexanal and β-ionone. Based on these common volatile compounds, these green teas may have green, floral, woody and citrus aroma. Information on volatile compounds of green teas from these areas was limited and only 2-Penten-1-ol has been reported from Vietnamese green tea [22]. The composition of 1-pentene-3-ol was 2-4 times higher in the sample from Vietnam than all other samples.

Green teas from Northeast Asia (China, Japan and Korea), the most common growers of green tea in international trade, typically had linalool, hexanal and nonanal, which will provide green and floral aromas. These compounds have been reported by others [36,43,44] from samples produced in the same region. Additionally, about half of the Chinese green teas had geraniol, benzaldehyde and jasmone. Those compounds previously have been reported from Chinese green tea [34,36,38] can provide floral and nutty aromas. About half of the Japanese green teas also had toluene and β-ionone. Toluene [40] and β-Ionone [12,44] have been reported in teas from Japan. Approximately 65% of Chinese green teas had β-ionone [25]. In this study, β-ionone was most commonly found in Japanese green tea (4 out of the 6 samples). Korean green teas also had benzeneethanol, benzaldehyde, and jasmone, which have been reported previously [7] and may provide floral, fruity, and nutty aromas. 

The parch-processed tea samples in Choi’s study [7] showed higher concentration of linalool when compared with the steam-processed samples, which is in agreement with our study. Linalool concentrations in Chinese and Korean samples generally were higher than Japanese samples. Chinese and Korean green teas are mostly roast-processed while Japanese green teas are steam-processed [13]. For instance *Longjing* (roast-processed Chinese green tea) contained linalool at much higher concentrations than *Sencha* (steam-processed Japanese green tea) [11]. China 7, which is a *Longjing* tea, had higher concentrations (0.55 ng/kg) than the *Sencha* teas from Japan (Japan 2 & 3 both at 0.27 ng/kg) (Table 3). In previous research *Longjing tea* had more geraniol than *Sencha tea* [11], which also was true for our study.

### 2.2. Principal Components Analysis of the Green Tea Samples

**Figure 1:**
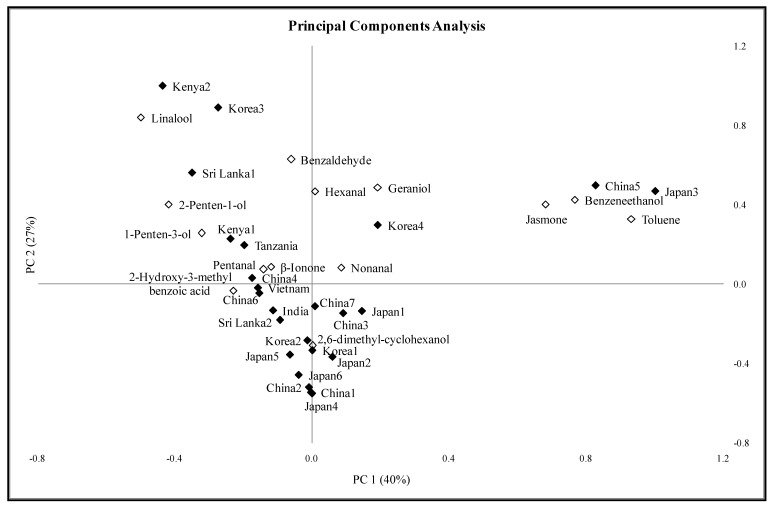
Principal components analysis biplot showing relationship between the green tea samples (♦) and the volatile compounds (◊).

Principal components analysis was conducted using only the mean concentration for the 14 compounds reported in Table 2. Because the volatile compounds found in less than four samples were not included in the PCA, many of the green teas could have unique flavors that would differentiate them from other teas in ways not shown on the PCA biplot (Figure 1). Principal components (PC) 1 and 2 explained 40% and 27% of the variation on the data, respectively. Toluene, benzeneethanol, jasmone, and linalool were the main vectors for PC 1. PC 2 accounted for linalool, benzaldehyde, geraniol and hexanal.

It is clear from the PCA map (Figure 1) that geography of the green teas in this study was not the primary differentiator based on volatile chemical composition. In fact, Japanese samples “map” in both the right-hand upper quadrant and the left-hand lower quadrant suggesting quite different volatile composition. Similarly, Korean samples occur in every quadrant showing that the composition of teas depends on many factors in addition to the inherent geographical characteristics of the growing location. Aspects such as the picking time, processing, packaging, and sample age all can impact the volatile composition.

Benzeneethanol, toluene, benzaldehyde, hexanal, nonanal, geraniol and jasmone were commonly present in China 5 and Japan 3 tea samples. Concentrations of toluene, benzeneethanol and jasmone in these two samples were higher than most of samples in the study. 

Kenya 2 and Korea 3 had a similar composition of aroma volatile compounds and were distinct from the rest of the samples. Concentrations of linalool were especially high in these 2 samples. Sri Lanka 1 had a comparable low volatile compound profile with Kenya 2 and Korea 3. In Korea 4 hexanal, jasmone and linalool were the main volatile compounds.

Eighteen samples, China 1, China 2, China 3, China 4, China 6, China 7, India, Japan 1, Japan 2, Japan 4, Japan 5, Japan 6, Kenya 1, Korea 1, Korea 2, Sri Lanka 2, Tanzania and Vietnam, were grouped together in the PCA biplot. These samples had two things in common: (1) the absence of some of the 14 volatile compounds that were semi-quantified in these tea samples, and (2) concentrations of the compounds in these samples (7 out of 14) that were much lower than other samples.

## 3. Experimental

### 3.1. Tea Samples

A total of 24 green teas from China (7), India (1), Japan (6), Kenya (2), Korea (4), Sri Lanka (2), Tanzania (1) and Vietnam (1) were selected for analysis (Table 4). Samples were selected from a pool of more than 130 samples and represent a range of green tea products from around the world. Although specific information other than origin was not known for every tea tested, it is likely that based on origin and price information, the teas represent different processing, different ages at picking, and different storage ages.

**Table 4:** List of 24 green tea samples.

	Label ^a^	Product name	Purchased from
1	China 1	*Star of China*	Imperialteagarden.com
2	China 2	*Gosancha*	XingHua Food Co., Ltd
3	China 3	*Imperial Hwangshan Maofeng*	Enjoyingtea.com
4	China 4	*Buddha’s Eyebrow*	Sevencups.com
5	China 5	*Myeng Ding Sweet Dew*	Sevencups.com
6	China 6	*Shanghai Special Gun Powder*	Shanghai Tiantan Int'l trading Co., Ltd.
7	China 7	*Longjing (Dragonwell)*	Adagio.com
8	India	*Assam Sewpur Estate*	Simpson & Vail Inc. (svtea.com)
9	Japan 1	*Tencha*	Harney & Sons (Harney.com)
10	Japan 2	*Honyama Sencha*	The Fragrant Leaf (thefragrantleaf.com)
11	Japan 3	*Sencha Overture*	Adagio.com
12	Japan 4	*Uji Mecha*	Itoen.com
13	Japan 5	*Uji Gyokuro*	Japanesegreenteaonline.com
14	Japan 6	*Inakacha*	Itoen.com
15	Kenya 1	*Kapchorua Green*	Culinaryteas.com
16	Kenya 2	*Kapchorua Green*	Barkingside.com
17	Korea 1	*Ssanggye Okchun*	Lotte Department Store, Busan, Korea
18	Korea 2	*Sulloc Sejac*	Donated by Amorepacific Co.
19	Korea 3	*Chungmyungcha*	Lotte Department Store, Busan, Korea
20	Korea 4	*Tobu Goku (Guyu)*	Tobu tea farm, Sunchon, Korea
21	Sri Lanka 1	*Iddalgashinna Estate Ceylon*	Uptontea.com
22	Sri Lanka 2	*Dumbara Curls*	Barkingside.com
23	Tanzania	*Tanzania Luponde Estate*	Simpson & Vail Inc. (svtea.com)
24	Vietnam	*Ha Giang Green Tea*	Thompsons (fineteas.com)

^a^ Label of samples is composed of ‘country of origin’ and a number when there were more than one sample from the same country.

### 3.2. Sample Preparation

Twelve grams of green tea were weighed and placed in a pre-warmed tea pot and 300 mL of 70 °C distilled water was added. The tea was brewed for 2 min and swirled 10 times clockwise while brewing. After 2 min, the tea was poured through a porcelain strainer into a pre-warmed porcelain bowl. This is similar to how consumers would brew green teas in many Asian countries [13,16] and recommended in a book intended for the public [14].

### 3.3. Solid-Phase Microextraction

For the extraction of the volatile compounds, 10 mL of each green tea sample was transferred into a 40 mL amber headspace vial (Supleco, Bellefonte, PA, USA). One micro liter of 1,3-dichlorobenezene (Sigma-Aldrich, Milwaukee, WI, USA) was added (concentration: 1 µg/Kg) as an internal standard for quantification of the volatile compounds from the samples. An octagonal, magnetic stir bar (Diameter 8 mm × length 13 mm; Fisher, Pittsburgh, PA, USA) and analytical grade sodium chloride (ca 3 g; Sigma-Aldrich) were added to the vial to help with the extraction. An open-center screw cap with a silicone/PTFE septum (22 mm diameter × 3.2 mm thickness, Supleco) was used to close the amber vial. Each sample was allowed to equilibrate at 60 °C in a Reacti-Therm™ heating block (Pierce Biotechnology, Inc., Rockford, IL, USA) for 5 min. Volatile odor compounds from the sample were extracted using a StableFlex 50/30 µm three phase (DVB/CAR/PDMS) SPME fiber (Supelco) at 60 °C for 20 min.

### 3.4. Gas Chromatograph-Mass Spectrometry

After extraction, the SPME fiber was retracted and the holder was moved to the splitless injection port of a 5890 Series II Gas Chromatography (Hewlett-Packard Co., Palo Alto, CA, USA) for manual injection. The fiber was desorbed for 5 min and the injection port was maintained at 225 °C. The volatiles were separated on an Rtx^®^-5 (Crossbond^®^ 5% diphenyl-95% dimethyl polysiloxane; 30 m length × 0.25 μm internal diameter × 0.25 mm film thickness) capillary column (Restek, Bellefonte, PA, USA). The temperature program for the separation was: 60 °C for 1 min, 18°C/min of ramp rate to 250 °C and held for 1 min. The total time was 12 min 34 s. The identification of the compounds was done using an HP 5890 Series II GC/HP 5972 mass selective detector (MSD, Hewlett-Packard Co., Palo Alto, CA, USA) with the following parameters: interface temperature, 250 °C; ionization energy, 70 eV; mass range, 33–350 a.m.u.; scan rate, 2.2 scans/s. Ultra high purity helium (AirGas, Westpoint, MS, USA) was used as a carrier gas at a constant flow rate of 0.96 mL/min. The mass spectra of the volatile compounds were compared using the Wiley138K Mass Spectral Database (Version B00.00, 1990; John Wiley and Sons, Inc., New York, NY, USA) for tentative identification. Volatile compounds were analyzed in triplicate for each sample. Concentrations for green tea volatile compounds were calculated and reported on the basis of the internal standard concentration.

### 3.5. Data Analysis

The mean concentrations (ng/kg) for the quantified volatile compounds were calculated and used for Principal Components Analysis (PCA; Unscrambler^®^ 9.7, CAMO Software Inc., Woodbridge, NJ, USA). A covariance matrix was used for the PCA. Use of appropriate principal component analysis has been reviewed by Yenket *et al*. [45].

## 4. Conclusions

The current study was an effort to understand volatile compounds in green tea as it typically might be brewed for consumption. Common volatile compounds in typically brewed green tea were tentatively identified. A number of compounds from prior research were not detected in this study most likely because of differences in the actual green tea samples used and because this study used a sample extraction and preparation method that was intended to mimic consumer preparation which had not been done before.

Linalool and hexanal were detected from almost all the green tea samples regardless of origin. In addition, green teas produced in Africa commonly had 1-penten-3-ol, 2-penten-1-ol and benzaldehyde. Green teas from Southeast Asia and the Indian subcontinent generally had nonanal. Green teas from Northeast Asia typically had nonanal and a few other compounds, such as benzene ethanol and jasmone not found in teas from other regions. Most volatile compounds found in our study have been reported elsewhere, although there were variations both qualitatively and quantitatively.

Further analysis is needed to determine compounds present in other green teas, ages of tea, and processing methods.
